# Calculating the optimal hematocrit under the constraint of constant cardiac power

**DOI:** 10.1038/s41598-021-83427-2

**Published:** 2021-02-16

**Authors:** Michal Sitina, Heiko Stark, Stefan Schuster

**Affiliations:** 1 Department of Anaesthesiology and Intensive Care, St. Anne’s University Hospital and International Clinical Research Center, Pekarska 53, 656 91 Brno, Czech Republic; 2 Department of Bioinformatics, Matthias Schleiden Institute, Friedrich Schiller University Jena, Ernst-Abbe-Platz 2, 07743 Jena, Germany; 3 Institute of Zoology and Evolutionary Research, Friedrich Schiller University Jena, Erbertstrasse 1, 07743 Jena, Germany

**Keywords:** Blood flow, Computational biophysics, Computational models, Computer modelling

## Abstract

In humans and higher animals, a trade-off between sufficiently high erythrocyte concentrations to bind oxygen and sufficiently low blood viscosity to allow rapid blood flow has been achieved during evolution. Optimal hematocrit theory has been successful in predicting hematocrit (HCT) values of about 0.3–0.5, in very good agreement with the normal values observed for humans and many animal species. However, according to those calculations, the optimal value should be independent of the mechanical load of the body. This is in contradiction to the exertional increase in HCT observed in some animals called natural blood dopers and to the illegal practice of blood boosting in high-performance sports. Here, we present a novel calculation to predict the optimal HCT value under the constraint of constant cardiac power and compare it to the optimal value obtained for constant driving pressure. We show that the optimal HCT under constant power ranges from 0.5 to 0.7, in agreement with observed values in natural blood dopers at exertion. We use this result to explain the tendency to better exertional performance at an increased HCT.

## Introduction

In the blood flow of humans and higher animals, a trade-off between sufficiently high erythrocyte concentration (known as hematocrit or HCT for short) to transport oxygen and sufficiently low blood viscosity to allow rapid flow has been achieved during evolution. Based on that trade-off, authors from our group calculated the optimal HCT value maximizing oxygen supply to be 0.3–0.5, which is in very good agreement with values observed in humans and many animal species^1,2^. The exact predicted value depends on the formula used to express viscosity in terms of HCT. Several other detailed theoretical modelling studies^3,4^ yield the optimal hematocrit value of about 0.4 as well. This shows that even a highly simplified model^1,2^ can lead to relevant results. An optimal hematocrit at rest of about 0.4 was also found in an experiment with dogs in which the hematocrit was varied by blood exchange^5^.

However, according to the calculations^1–4^, the optimal value should be independent of the level of exertion. This is in contradiction with the observation in endurance runner animals like dogs or horses, which are called natural blood dopers^6^ because they increase their hematocrit at exertion via expulsion of concentrated blood from the spleen. Another contradiction comes from the prohibited practice of blood boosting in high-performance sports. With the use of blood boosting, several outstanding records were set and many studies have demonstrated its positive performance effect^7–19^. 

In other studies^20^, however, no increase in performance was observed. Even placebo effects have been mentioned to solve this “hematocrit paradox” and the topic remains controversial^6^. A drawback of the idea about a positive effect of an increased hematocrit is the absence of a clear theoretical explanation: why should the increased hematocrit promote performance, when the theoretical models^1,3,4^ predict the optimum to be at normal values even at exertion. In this work, we introduce the new optimization constraint of constant cardiac power and suggest that its use provides the missing theoretical explanation. That constraint implies that the product of cardiac output per time unit and arterial blood pressure remains constant.

Before performing the calculation of the optimal hematocrit under the constraint of constant cardiac power, we recapitulate the calculation under constant perfusion pressure. We discuss both optimization approaches in a broader context of cardiovascular physiology. The former constraint of constant perfusion pressure was previously used in the theoretical models mentioned above^1,3,4^. We will discuss here its relevance for the resting condition. At exertion, however, the body switches to performance with a resulting change in blood flow regulation. We justify here that the latter constraint of constant cardiac power newly proposed here is more relevant for this exertional condition than the former one. We show that the resulting optimal hematocrit is higher than the normal value. With this result, we explain the tendency to better exertional physical performance at an increased hematocrit, at least over shorter periods.

## Methods and results

### Basic equations

According to the Hagen–Poiseuille law^21^, the flow *Q* through a tube of length $l$ and radius $r$ under the pressure difference $\Delta p$ (alternatively called perfusion pressure or driving pressure) is given by

1 $${\text{Q}}=\frac{\pi {r}^{4}\Delta p}{8l\eta \left(\varphi \right)}$$

where $\eta \left(\varphi \right)$ denotes the fluid viscosity, which is, in the case of blood, a function of the hematocrit $\varphi$.

For our calculations, it is only relevant that the flow is proportional to $\Delta p$ and inversely proportional to the viscosity *η*. This also holds for an ideal flow in a vessel with elliptical or rectangular cross-section^22^ or the flow through porous media^23^. Thus, Eq. (1) can be rewritten as

2 $${\text{Q}}=K\frac{\Delta p}{\eta \left(\varphi \right)}$$

with some constant $K$ dependent only on the tube geometry. This relationship can be generalized for the whole circulation or segments of circulation^24,25^, with *Q* being the total flow through the circulation per time unit (i.e. cardiac output) and $\Delta p$ the pressure difference between both ends of the circulation (i.e. the difference between the mean arterial pressure and the pressure in the right atrium of the heart). The relationship is valid for subsegments of circulation as well, such as for the venous part of circulation. Then $\Delta p$ corresponds to the difference between capillary pressure and right atrial pressure.

**Figure 1:**
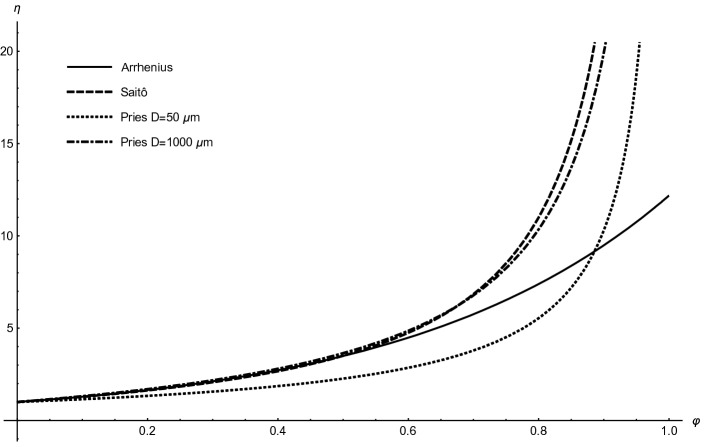
Dependencies of the (relative) viscosity $\eta$ on the hematocrit $\varphi$ according to several selected formulas from Table 1.

In the following sections, we use several relationships between the hematocrit and blood viscosity that are summarized in Table 1. Some of these relationships are illustrated in Fig. 1. $\eta$_0_ denotes the viscosity of the particle-free liquid, which is blood plasma in our case. The hematocrit $\varphi$ represents the volume fraction of erythrocytes in the blood, $\varphi$_m_ is the maximum possible volume fraction of erythrocytes (maximal packing density). In case of stiff spherical particles, for example, $\varphi$_m_ equals 74%. The formulas are discussed in Stark and Schuster^1,2^.

**Table 1:** Relationships between blood viscosity $\eta$ and the hematocrit $\varphi .$

Author	Formula	
Einstein^48,49^	$\eta ={\eta }_{0}\left(1+2.5\varphi \right)$	(3)
Gillespie^50^	$\eta ={\eta }_{0}\frac{1+\varphi /2}{{\left(1-\varphi \right)}^{2}}$	(4)
Krieger and Dougherty^51^	$\eta =\frac{{\eta }_{0}}{{\left(1-\varphi /{\varphi }_{m}\right)}^{2.5{\varphi }_{m}}}$	(5)
Arrhenius^52^	$\eta ={\eta }_{0}{e}^{2.5\varphi }$	(6)
Saitô^27^	$\eta ={\eta }_{0}\left(1+2.5\frac{\varphi }{1-\varphi }\right)$	(7)
Quemada^53^	$\eta =\frac{{\eta }_{0}}{{\left(1-\varphi /{\varphi }_{m}\right)}^{2}}$	(8)
Mooney^54^	$\eta ={\eta }_{0}{e}^{\frac{2.5\varphi }{1-\varphi /{\varphi }_{m}}}$	(9)

*η*_0_ is the viscosity of blood plasma,* φ*_m_ is the maximum possible volume fraction of erythrocytes.

Specially for blood, a semi-empirical, more precise formula

10 $$\eta \left(D,\varphi \right)={\eta }_{0}\left(1+\frac{\left(2.2-2.44{e}^{-0.06{D}^{\mathrm{0,645}}}+220{e}^{-1.3D}\right)\left[-1+{\left(1-\varphi \right)}^{w+\left(w-1\right)\left(0.8+{e}^{-0.075D}\right)}\right]}{{0.55}^{w+\left(w-1\right)\left(0.8+{e}^{-0.075D}\right)}-1}\right)$$

was developed by Pries^26^, where *D* represents the tube diameter (in μm) and the factor $w=1/\left(1+{D}^{12}/{10}^{11}\right)$. In case of a rapid flow in a small vessel, the erythrocytes, as very large ‘particles’, are concentrated in the middle of the vessel, which decreases the effective blood viscosity (known as Fåhraeus–Lindqvist effect^1,2^). Therefore, the effective viscosity depends not only on the hematocrit $\varphi$, but also on the vessel diameter *D*. The Pries formula takes account of this behaviour and can be regarded as most reliable. In small vessels, viscosity is less sensitive to hematocrit, which is reflected in the curve for small vessel diameters (50 μm) in Fig. 1, lying below that for large diameters.

The oxygen content in one litre of fully saturated blood, ${C}_{ox}$*,* is proportional to the hematocrit $\varphi$, with the proportionality constant $k$

11 $${C}_{ox}=k\varphi $$

The whole organism oxygen supply, further denoted as ${\mathrm{J}}_{\mathrm{ox}}$ an be expressed as

12 $${J}_{ox}={C}_{ox}Q=k\varphi Q$$

In the following sections, two constrained optimization models of oxygen supply will be described—a model with a constraint of constant driving pressure and a model with a constraint of constant cardiac power.

### Optimization of oxygen supply under constant driving pressure

Optimization with constant driving pressure is similar to the optimization performed by our group formerly^1,2^. Only the main ideas of that work will be summarized in this section.

With the substitution of *Q* in Eq. (12) from Eq. (2) we obtain ${J}_{ox}$ as a function of $\Delta p$ and $\varphi$

13 $${J}_{ox}=k{K\varphi \frac{\Delta p}{\eta \left(\varphi \right)}=K}{^{\prime}}\Delta p\frac{\varphi }{\eta \left(\varphi \right)}$$

The product $kK$ was replaced with a new constant ${K}{^{\prime}}$.

For a given driving pressure $\Delta p,$ the oxygen supply ${J}_{ox}$ achieves maximum at

14 $$0={\left.\frac{\partial {J}_{ox}}{\partial \varphi }\right|}_{\Delta p}=\frac{{K}{^{\prime}}\Delta p}{{\eta }^{2}\left(\varphi \right)}\left(\eta \left(\varphi \right)-\varphi {\eta }{^{\prime}}\left(\varphi \right)\right)$$

which results in a condition for the optimal hematocrit ${\varphi }_{max}^{\Delta p}$

15 $${\varphi }_{max}^{\Delta p}=\frac{\eta \left({\varphi }_{max}^{\Delta p}\right)}{{\eta }{^{\prime}}\left({\varphi }_{max}^{\Delta p}\right)}$$

That this leads to a maximum can be confirmed with the negative value of the second derivative. Equation (15) has not yet been derived in our previous papers^1,2^.

**Figure 2:**
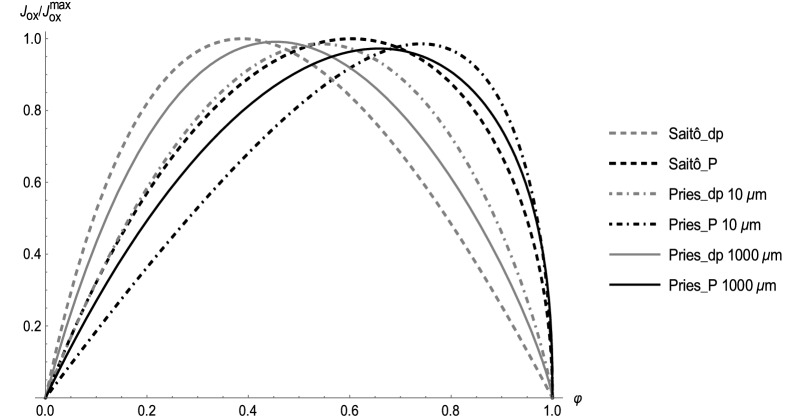
Dependence of ${J}_{ox}$ on $\varphi$ for several selected formulas for $\eta \left(\varphi \right).$ Grey curves—calculation under constant driving pressure (_dp), black curves—calculation under constant cardiac power (_P). ${J}_{ox}$ is plotted as a fraction of maximal ${J}_{ox}$ of the respective curve.

**Figure 3:**
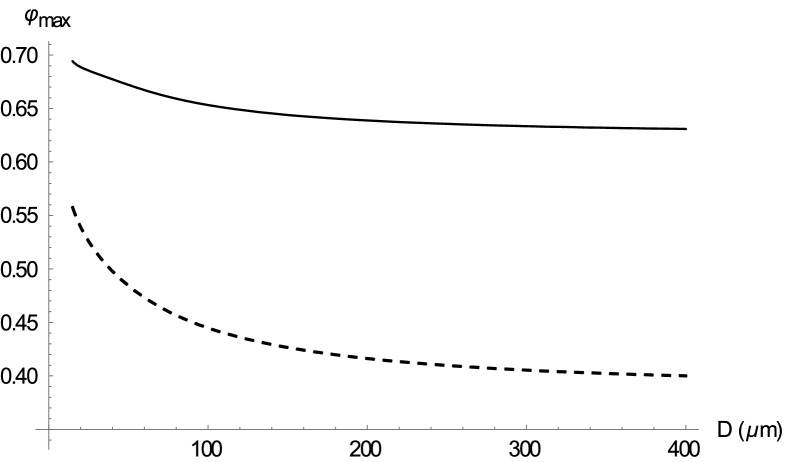
Dependence of the optimal hematocrit ${\varphi }_{max}$ on the vessel diameter D, based on the Pries formula for the optimization under constant driving pressure (dashed curve) and constant cardiac power (solid curve).

The different optimal values ${\varphi }_{max}^{\Delta p}$ following from various formulas for $\eta \left(\varphi \right)$ are given in Table 2 (left column), ranging mostly from 0.3 to 0.4. Selected plots of ${J}_{ox}$ as a function of $\varphi$ under constant driving pressure are shown in Fig. 2. Figure 3 illustrates the influence of the vessel diameter, based on the Pries formula. For small vessels, the lower effective viscosity results in a higher optimal hematocrit, reaching the optimal value of more than 0.5 for vessel diameters below 50 μm. For large diameters the Pries formula provides approximately the same optimal value of 0.39 as Saitô’s formula^1,27^. It is worth noting that the optimization in the detailed model of Farutin et al.^3^ was also performed under constant driving pressure and yields a similar optimal hematocrit value of about 0.4.

**Table 2:** Comparison of optimal values of the hematocrit, optimized under constant driving pressure (left column) and constant cardiac power (right column).

Viscosity formula	${\varphi }_{max}^{\Delta p}$	${\varphi }_{max}^{P}$
Mooney	0.234	0.344
Quemada	0.333	0.505
Krieger–Dougherty	0.286	0.491
Gillespie	0.302	0.474
Saitô	0.387	0.603
Arrhenius	0.400	0.800
Pries D = 10 μm	0.608	0.731
Pries D = 1000 μm	0.393	0.627

### Optimization of oxygen supply under constant cardiac power

Now we modify the circulation model so that the physical power of the heart *P* rather than the driving pressure $\Delta p$ remains constant.

The physical power *P* of the heart is defined as the work performed by the heart in a unit time. Thus,

16 $$P=\frac{dW}{dt}=\frac{dV\Delta p}{dt}=Q\Delta p$$

where *W* is the work of the heart, *V* denotes the ejected blood volume and dV/dt is the cardiac output.

With a substitution from Eq. (2) we obtain

17 $$P=Q\Delta p=K\frac{{\left(\Delta p\right)}^{2}}{\eta \left(\varphi \right)}$$

Substituting $\Delta p$ in Eq. (2) we obtain $Q$ as a function of $P$ and $\varphi$

18 $$Q=K\frac{\Delta p}{\eta \left(\varphi \right)}=\sqrt{K}\frac{\sqrt{P}}{\sqrt{\eta \left(\varphi \right)}}$$

For a given cardiac power *P* the blood flow depends on the inverse square root of viscosity, contrary to just inverse linear dependence in case of constant driving pressure, as follows from Eq. (2). This makes the blood flow less dependent on viscosity. A lower sensitivity of blood flow to hematocrit at exertion was indeed found in experiment^28^.

For ${J}_{ox}$, it follows

19 $${J}_{ox}=k\varphi Q={K}{^{\prime}}\sqrt{P}\frac{\varphi }{\sqrt{\eta \left(\varphi \right)}}$$

where the constant terms were replaced with a new constant ${K}{^{\prime}}.$

For a given cardiac power *P*, the oxygen supply ${J}_{ox}$ achieves maximum at

20 $$0={\left.\frac{\partial {\text{Jox}}}{\partial \varphi }\right|}_{P}=\frac{{K}^{{^{\prime}}{^{\prime}}}\sqrt{P}}{\eta \left(\varphi \right)}\left(\sqrt{\eta \left(\varphi \right)}-\varphi \frac{{\eta }{^{\prime}}\left(\varphi \right)}{2\sqrt{\eta \left(\varphi \right)}}\right)$$

which results in a condition for ${\varphi }_{max}^{P}$

21 $${\varphi }_{max}^{P}=\frac{2\eta \left({\varphi }_{max}^{P}\right)}{{\eta }{^{\prime}}\left({\varphi }_{max}^{P}\right)}$$

Hence, the ${\varphi }_{max}^{P}$ represents the hematocrit that maximizes the oxygen supply under a given cardiac power. Again, this can be confirmed with the negative value of the second derivative.

As in the previous subsection, various formulas for $\eta \left(\varphi \right)$ provide different optimal hematocrit values ${\varphi }_{max}^{P}$, given in Table 2 (right column), ranging mostly from 0.5 to 0.7. Selected plots of ${J}_{ox}$ as a function of $\varphi$ at a given cardiac power are shown in Fig. 2. Figure 3 illustrates the influence of vessel diameter, based on the Pries formula. For small vessels the optimal hematocrit increases up to 0.7, for big vessels we obtain an optimal value of around 0.6, just as from Saitô’s formula^27^.

### Relationship between ${\varphi }_{max}^{P}$ and ${\varphi }_{max}^{\Delta p}$

As can be seen in Table 2, the optimal hematocrit under constant cardiac power is always higher than the optimal hematocrit under constant driving pressure, that is

22 $${\varphi }_{max}^{P}>{\varphi }_{max}^{\Delta p}$$

This can be proved in a general way. The implicit Eqs. (15) and (21) can be rearranged to

23 $${\eta }{^{\prime}}\left({\varphi }_{max}^{\Delta p}\right)=\frac{\eta \left({\varphi }_{max}^{\Delta p}\right)}{{\varphi }_{max}^{\Delta p}}$$

24 $${\eta }{^{\prime}}\left({\varphi }_{max}^{P}\right)=2\frac{\eta \left({\varphi }_{max}^{P}\right)}{{\varphi }_{max}^{P}}$$

**Figure 4:**
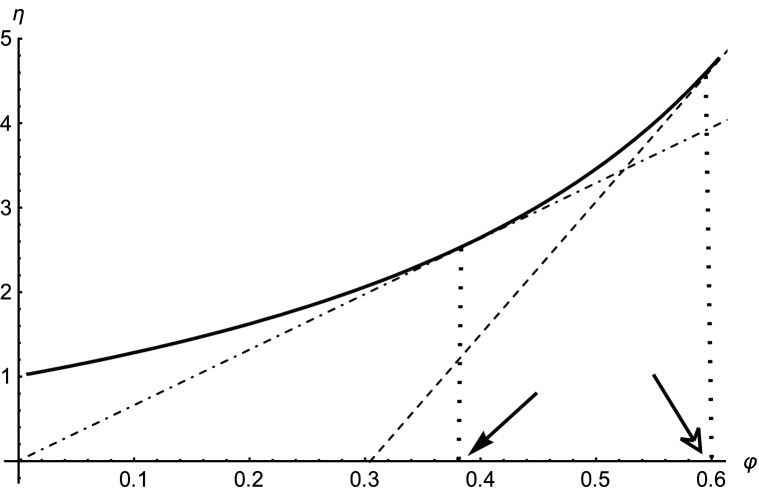
Geometric interpretation of ${\varphi }_{max}^{\Delta p}$ (full arrow) and ${\varphi }_{max}^{P}$ (empty arrow). Solid curve—function $\eta \left(\varphi \right)$, dash-dotted line—tangent in the point ${\varphi }_{max}^{\Delta p}$, dashed line—tangent in the point ${\varphi }_{max}^{P}$. For further explanations, see text.

These equations have the following geometric interpretation (Fig. 4). The tangent to the function $\eta \left(\varphi \right)$ in the point ${\varphi }_{max}^{\Delta p}$ (dash-dotted line) intersects the abscissa in the origin of coordinates. The tangent in the point ${\varphi }_{max}^{P}$ (dashed line) intersects the x-axis in the point ${\varphi }_{max}^{P}/2$. As the function $\eta \left(\varphi \right)$ is positive, monotonic increasing and strictly convex, inequality (22) must always hold.

Intuitively, the higher optimal value can be explained with a weaker dependence of blood flow on the viscosity at given cardiac power—a dependence on the inverse square root of viscosity contrary to the inverse proportionality, as noted above.

Since Arrhenius’ formula is an exponential function, a comparison of Eqs. (15) and (21) shows that the optimal value ${\varphi }_{max}^{P}$ is double as high as ${\varphi }_{max}^{\Delta p}$ when that formula is used. For the other functions, that is not the case.

## Discussion

We have presented two models of global oxygen supply, which differ in their constraints—notably constant driving pressure and constant cardiac power. We have shown by a novel calculation that the oxygen supply in the latter case achieves maximum at higher hematocrit values. More precisely, the optimal hematocrit under the constraint of constant driving pressure ranges from 0.3 to 0.5 and under constant cardiac power from 0.5 to 0.7.

It is worth mentioning that both optimization problems under study can be formulated as different but equivalent problems, which lead to the same optimal hematocrit values but have a more intuitive physical interpretation. The equivalence can easily be shown by rearrangement of the equations or by the method of Lagrange multipliers. Maximizing oxygen supply under constant driving pressure is equivalent to minimizing the driving pressure under constant oxygen supply. An analogous statement holds for maximizing oxygen supply under constant cardiac power.

**Figure 5:**
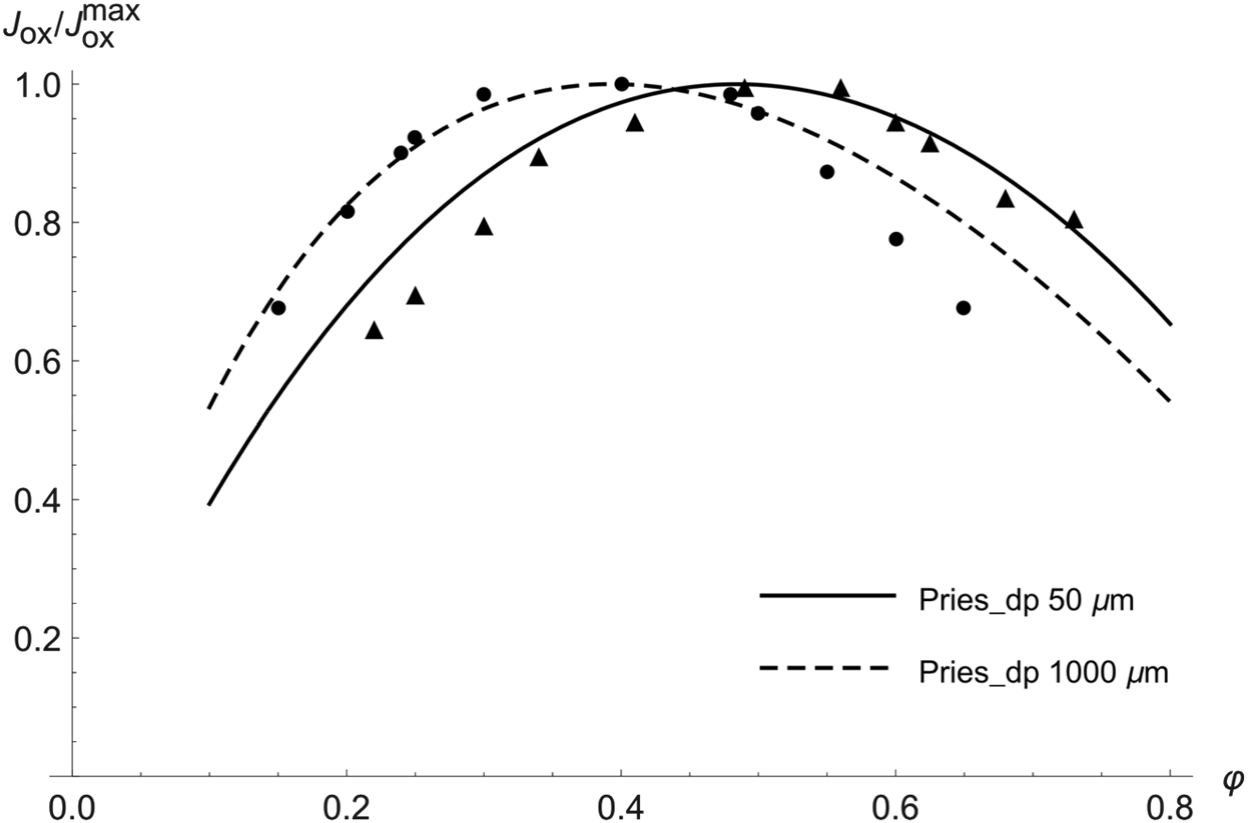
Comparison of normalized experimental data from the studies by Gaehtgens^36^ and Guyton^5^ with our model predictions under the constraint of constant driving pressure—dependence of oxygen supply on hematocrit. Solid line—model prediction using the Pries formula for diameter of 50 μm, dashed line—model prediction using the Pries formula for diameter of 150 μm, triangles—experimental data for working skeletal muscle ( taken from Fig. 7 of^36^), circles—experimental data in the resting situation (taken from Fig. 3 of^5^).

We will now discuss the physiological relevance of the calculated results and their relation to other studies. The first model with the constant driving pressure yields optimal hematocrit values near the normal values of many animals, such as cat, pig, orangutan, chimpanzee and killer whale^1^. Guyton et al.^5^ changed, in experiment, the hematocrit in dogs at rest with the use of blood transfusion without any change in blood volume. The blood pressure, mean circulatory filling pressure and central venous pressure and, thus, also the driving pressure remained almost unchanged during the experiment, resembling the constant driving pressure constraint of our first model. The highest oxygen supply was found at the hematocrit of 0.4, just as predicted by the model. As shown by Guyton et al.^29,30^, the filling and resistance of the venous system rather than heart performance and arteriolar resistance determine the cardiac output at rest. Using the Pries formula (Fig. 3, dashed curve), the optimal value of 0.4 applies to relatively large vessel diameters, corresponding very well with the large diameter of venules and veins, but not of arterioles. Moreover, our first model provides an exact prediction not only of the optimal value but also of the blood flow at other hematocrit values, as is documented in Fig. 5 (grey points) by the agreement of experimental data with our prediction. Just the prediction for very low or very high hematocrit values agrees with experimental data less exactly, which could be explained by compensatory vasodilation at extreme values of hematocrit, not considered in our model. Thus, the first model provides a good explanation of the optimal hematocrit value at rest.

Different from the situation at rest are the circulatory conditions at an extreme exercise, e.g. during high-performance sport. It was shown that the heart achieves its maximum power in that case and is a limiting factor for maximum oxygen uptake in tissues^31^. Under the plausible assumption that a maximal cardiac power implies that it remains constant over a certain period, the optimization at an extreme exercise corresponds to our second model. Our model then predicts the optimal hematocrit to range from 0.5 to 0.7, which is in good agreement with 0.58 found in the study of Schuler et al.^17^ or 0.6 of hunted horses as an example of animals called natural blood dopers^6^.

Thus, the second model with the constant cardiac power constraint could provide the missing theoretical explanation why the optimal hematocrit should be increased at an extreme exertion, although the normal hematocrit is optimal at rest. At an extreme exertion, but not at rest, the energy output of the heart, which equals the energy loss in the circulation, is the limiting factor for the performance. To minimize energy loss, the hematocrit must be higher than normal, as follows from the equivalent problem of minimizing cardiac power under constant oxygen supply mentioned above. Most of the energy is lost on the level of small arterioles with a diameter of 20–100 μm^32,33^. For this diameter, the calculation based on the Pries formula provides the optimal value of 0.65, which is very close to the experimental values of 0.58 or 0.6 mentioned above.

There are, however, several drawbacks of the presented explanation. Firstly, the cardiac power, i.e. the mechanical energy output from the heart, is not necessarily the most relevant constraint at exertion. Another limiting factor, which becomes relevant at exertion, could be the energy (or oxygen) consumption of the heart, which is mainly determined by the coronary perfusion and heart metabolism. If the heart work efficiency, i.e. the percentage of energy consumption converted to mechanical work, was constant, cardiac energy output and energy consumption would be equivalent constraints in our second model. It was shown, however, that the heart work efficiency markedly declines with rising arterial blood pressure^34^, accompanying the increased hematocrit as a compensation of increased viscosity. Thus, our second model describes an idealized, extreme case. The true optimal value is likely to be situated between both extreme models. An interesting analogy is a bicycle with a gear shift. When using a high gear, the pedals need to be moved with low velocity only, yet with high force. There is an upper bound on the force generated by the leg muscles and also by the heart. It is then easier to switch to a lower gear, and so it is to pump the blood at a lower hematocrit. The danger of extremely high blood pressure upon blood doping can be documented by the death of 18 Dutch and Belgian cyclists from 1987 to 1990^35^.

**Figure 6:**
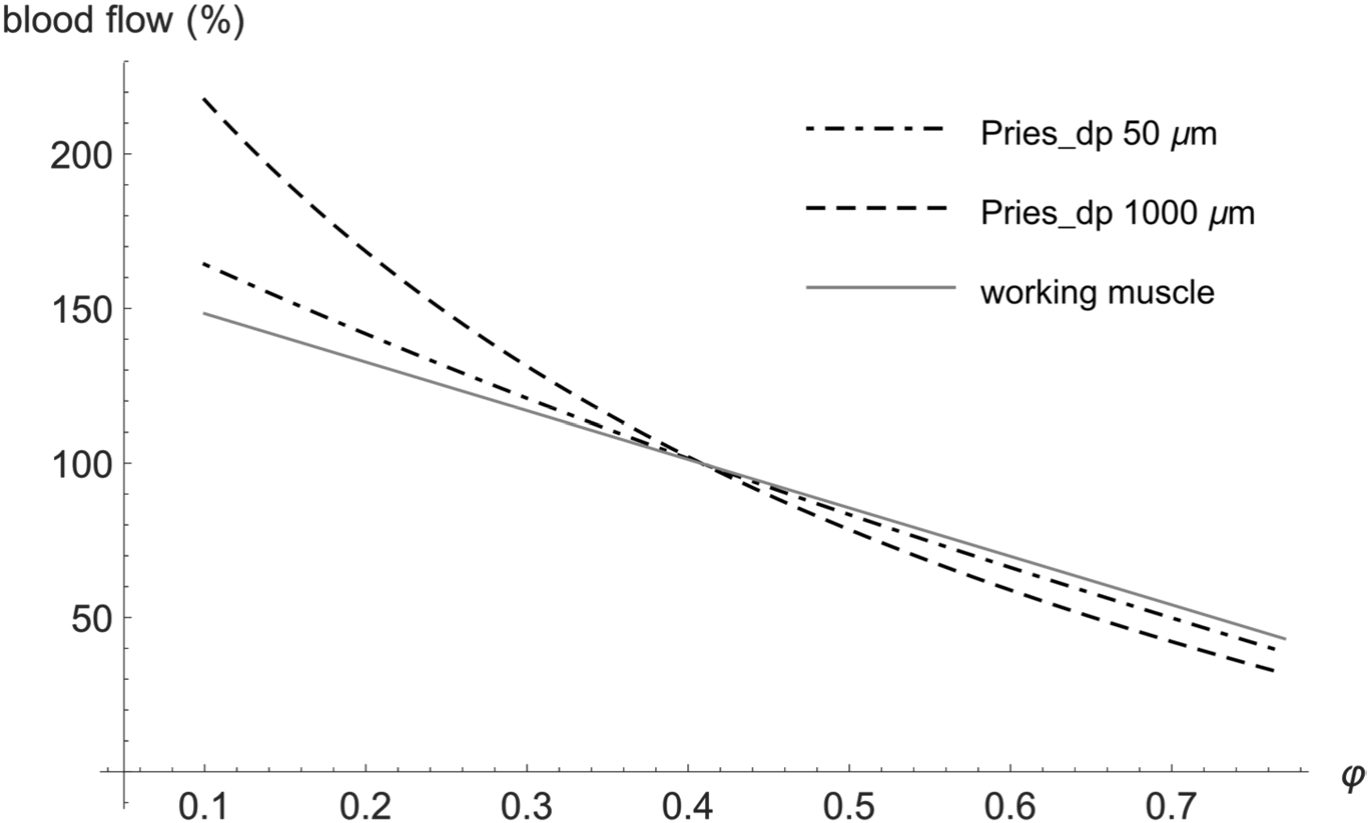
Comparison of experimental data from the study by Gaehtgens^36^ with our model predictions under the constraint of constant perfusion pressure—dependence of relative blood flow on hematocrit. Blood flow of 100% corresponds to a hematocrit of 0.4 for all curves. Dashed line—model prediction using the Pries formula for a diameter of 1000 μm, dot-dashed line—model prediction using the Pries formula for a diameter of 50 μm, solid grey line—experimental data for working skeletal muscle (fitted in^36^ by the linear function *Q* = 164–157 $\varphi$).

Secondly, Gaehtgens et al.^36^ studied the dependence of oxygen supply on hematocrit in an isolated working skeletal muscle of a dog that was artificially perfused with constant perfusion pressure. They found optimal hematocrit values of 0.5–0.6 during exercise and 0.3–0.4 at rest. Contrary to our models, both the resting and exertional optimal values were determined under the constraint of constant perfusion pressure. It could seem, therefore, that no additional new constraint is necessary to explain the increased optimal hematocrit at exertion. Actually, we can explain Gaehtgens’ results with our first model using the Pries formula under the constraint of constant perfusion pressure as well. In Fig. 6 we compare experimental data—the dependence of relative blood flow on hematocrit—taken from Fig. 6 of Gaehtgens’ study^36^ with predictions of our first model. For the calculations, we used the Pries formula for small (50 μm) and large (1000 μm) vessels. That formula includes the influence of the vessel diameter on the effective blood viscosity (i.e. the Fåhraeus–Lindqvist effect). As seen from Fig. 5, our predictions for small vessels are in very good agreement with experimental data for working skeletal muscle in a broad range of hematocrit. Similarly, Fig. 5 shows a very good agreement between the normalized experimental data—the dependence of relative oxygen supply on hematocrit—for the working muscle (black points) taken from Fig. 7 of^36^ and our prediction using the Pries formula for small vessel diameter. Both the model and experiment provide the same optimal hematocrit value of 0.5, which is, however, slightly lower than the values 0.58 or 0.6 mentioned above. The agreement of our predictions for narrow vessels is somewhat surprising, though, because arterioles are supposed to be fully dilated at exertion, rather than being very narrow. We explain this with a mechanical effect of muscle contractions that may compress arterioles and propel blood through the microcirculation and mitigate in this way the influence of hematocrit on viscosity^28^. In a similar way, the Fåhraeus–Lindqvist effect mitigates the influence of hematocrit in very small vessels.

As noted above, the model under the constant driving pressure constraint for narrow vessels as well as Gaehtgens’s study^36^ provide higher than normal optimal hematocrit values, but still lower than the values measured at exertion. Furthermore, we maintain that, from a physiological perspective, the constraint of constant driving pressure does not correspond well with the heart as a limiting factor at extreme exertion, as described above. Therefore, we consider the second model under constant cardiac power as a more valid explanation of the positive effect of the increased hematocrit at exertion, at least as a tendency.

It is worth mentioning that both models are rather simple and do not consider many complex effects such as deformation and aggregation of erythrocytes, vasodilation, oxygen consumption of the heart, effect of temperature, elasticity of blood vessels, pulsatility of the blood flow or non-Newtonian behaviour of the blood, which is, however, partially considered by using Pries’ formula (Fåhraeus–Lindqvist effect). Despite these caveats, the very good agreement of our predictions with experimental data (Fig. 5) support our opinion that the most relevant factors are taken into account. Moreover, the much more detailed model of Farutin^3^ yields, in the case of the constant driving pressure constraint, the same optimal value as our simplified model. In future studies, it can be of interest to extend the model by including such effects. In the following, we will shortly comment some of the neglected effects.

An important question is the effect of temperature. Viscosity decreases with increasing temperature. However, this effect is mediated by the viscosity of the pure liquid, *η*_0_ rather than by the other terms in the formulas for the function *η*(*ϕ*). According to the two models presented above, the optimal Hct should not depend on temperature because *η*_0_ drops out in the calculation. This is supported by the observation that the hematocrit values are quite robust against temperature changes^37,38^.

However, poikilothermic animals have to cope with changing temperatures and to avoid a harmfully viscous blood in the cold. Thus, it may be beneficial for them to have a lower HCT^2^. This view is supported by the observation that many fish species, especially cartilaginous fish, have HCT values of 20–30%^39^.

The effect of temperature on blood viscosity could be relevant at extreme exertion, where the core temperature of human body can increase up to 40 °C, corresponding to the blood viscosity decrease of about 10%^40^. What determines the optimal hematocrit value, however, is not the absolute value of the blood viscosity, but the shape of the viscosity-hematocrit curve. As can be seen in Fig. 1A–C in^41^, the shape of these curves does not change substantially with temperature.

Flow pulsatility could be significant for our second model when dealing with arterioles. In these vessels, the pulsatility index is about 80%^42^. That means that the maximal flow as well as shear rate in arterioles can be up to twice as high as the minimal one. Figure 1C in^41^ gives the viscosity-hematocrit curves for different shear rates in the range of 45–450 s^−1^. These curves, for the temperature range relevant for humans, are very similar. For the range of arteriolar flow pulses of the factor 2, the differences are negligible. Moreover, the shape of those curves is even more similar. We think, therefore, that the use of a mean flow velocity instead of the pulsatility flow does not introduce any considerable error.

We model vessels as rigid tubes, although they are elastic. In arterioles, the pressure pulsations with subsequent vessel diameter pulsations are less than 25% of the mean value^43^. Therefore, we ignore this effect and consider the rigid tube model a relevant simplification.

Finally, we operate with hematocrit instead of the concentration of hemoglobin, which binds oxygen. We assume, however, a constant concentration of hemoglobin in erythrocytes. Then, both hematocrit and hemoglobin are equivalent proxies of the blood oxygen binding capacity. And it is the hematocrit rather than hemoglobin itself that determines blood viscosity. A more complex model could optimize the size of erythrocytes, concentration of hemoglobin inside erythrocytes and hematocrit at the same time, since the hemoglobin concentration indeed differs between several species, for example, between rays, sharks, and teleost fish^39^. An interesting question is: why did evolution set the hematocrit to the optimal value at rest and not to that at exertion? Probably the long-term risks of an increased hematocrit (e.g. increased blood pressure with a consequent chronic heart failure or increased risk of a thrombosis) may have outweighed the short-term advantages during an extreme exertion. As mentioned above, some animal species, such as dogs and horses, called natural blood dopers, make use of the high hematocrit during exertion, without facing a long-term risk. Interestingly, both dogs and horses are companions of man used for hunting for a long time and could be selected for blood doping. Alternatively, prehistoric people could domesticate these animals just because of this ability. Interestingly, the top aerobic speed (speed of endurance running) of both animals is about 40 km/h and, thus, maximum among mammals^44^. 

## Conclusions

Our calculations predict optimal hematocrit values of 0.5–0.7 at intense exertion, which is in good agreement with experimental observations for natural blood dopers such as horses. Taking into account several features of exercise physiology, we consider constant cardiac power a reasonable constraint that explains the higher optimal value of hemoglobin at exertion than at rest. Moreover, the presented analysis shows that the optimal hematocrit is likely to depend on conditions, so that no unique value can be indicated.

The above calculations can be promising for future applications in personalized medicine. For example, for the therapy of patients with limited cardiac power, as in the cardiogenic shock^45,46^, calculating the optimal hematocrit value could be helpful. The treatment goal for such patients would be to supply a given amount of oxygen, but to minimize necessary cardiac power. A special application may concern the treatment of heart diseases in highlanders of different populations^47^. 

## Abbreviations

*C*_*ox*_: Oxygen content in one litre of full saturated blood
*D*: Tube diameter
$$\varphi $$: Hematocrit (HCT)
${\varphi }_{m}$: Maximum possible HCT
${\varphi }_{max}^{\Delta p}$: Optimal HCT under constant driving pressure
${\varphi }_{max}^{P}$: Optimal HCT under constant cardiac power
${\mathrm{J}}_{\mathrm{ox}}$: Oxygen supply to tissues
*l*: Length of a tube
$$\eta $$: Blood viscosity
*η*_*0*_: Viscosity of blood plasma
$\Delta p$: Pressure difference/driving pressure
*P*: Heart power (work performed by the heart in a unit time)
*Q*: Blood flow
*r*: Tube radius
